# Combating air pollution significantly reduced air mercury concentrations in China

**DOI:** 10.1093/nsr/nwae264

**Published:** 2024-07-29

**Authors:** Xinbin Feng, Xuewu Fu, Hui Zhang, Xun Wang, Longyu Jia, Leiming Zhang, Che-Jen Lin, Jen-How Huang, Kaiyun Liu, Shuxiao Wang

**Affiliations:** State Key Laboratory of Environmental Geochemistry, Institute of Geochemistry, Chinese Academy of Sciences, Guiyang 550081, China; University of Chinese Academy of Sciences, Beijing 100049, China; State Key Laboratory of Environmental Geochemistry, Institute of Geochemistry, Chinese Academy of Sciences, Guiyang 550081, China; State Key Laboratory of Environmental Geochemistry, Institute of Geochemistry, Chinese Academy of Sciences, Guiyang 550081, China; State Key Laboratory of Environmental Geochemistry, Institute of Geochemistry, Chinese Academy of Sciences, Guiyang 550081, China; State Key Laboratory of Environmental Geochemistry, Institute of Geochemistry, Chinese Academy of Sciences, Guiyang 550081, China; University of Chinese Academy of Sciences, Beijing 100049, China; Air Quality Research Division, Science and Technology Branch, Environment and Climate Change Canada, Toronto M3H 5T4, Canada; Center for Advances in Water and Air Quality, Lamar University, Beaumont, TX 77710, USA; State Key Laboratory of Environmental Geochemistry, Institute of Geochemistry, Chinese Academy of Sciences, Guiyang 550081, China; State Key Joint Laboratory of Environment Simulation and Pollution Control, School of Environment, Tsinghua University, Beijing 100084, China; College of Environmental Science and Engineering, North China Electric Power University, Beijing 102206, China; State Key Joint Laboratory of Environment Simulation and Pollution Control, School of Environment, Tsinghua University, Beijing 100084, China

**Keywords:** atmospheric mercury, long-term trend, anthropogenic emission reductions, co-benefits of clean air action

## Abstract

In the past decade, China has motivated proactive emission control measures that have successfully reduced emissions of many air pollutants. For atmospheric mercury, which is a globally transported neurotoxin, much less is known about the long-term changes in its concentrations and anthropogenic emissions in China. In this study, over a decade of continuous observations at four Chinese sites show that gaseous elemental mercury (GEM) concentrations continuously increased until the early 2010s, followed by significant declines at rates of 1.8%–6.1% yr^−1^ until 2022. The GEM decline from 2013 to 2022 (by 38.6% ± 12.7%) coincided with the decreasing concentrations of criteria air pollutants in China and were larger than those observed elsewhere in the northern hemisphere (5.7%–14.2%). The co-benefits of emission control measures contributed to the reduced anthropogenic Hg emissions and led to the GEM decline in China. We estimated that anthropogenic GEM emissions in China were reduced by 38%–50% (116–151 tons) from 2013 to 2022 using the machine-learning and relationship models.

## Teaser

This study reports the world’s largest declines in atmospheric mercury concentrations in China since early 2010s, reflecting the co-benefits of China’s Clean Air Action on reducing the domestic anthropogenic mercury emissions.

## INTRODUCTION

China is the largest anthropogenic source region of major air pollutants due to rapid economic growth in the past two decades, contributing 20%–33% of the total amount of pollutants (e.g. mercury (Hg), sulfur dioxide (SO_2_), nitrogen oxides (NO*_x_*), carbon monoxide (CO), black carbon and organic carbon) emitted worldwide [1,2]. To protect human health, the Chinese government has been implementing comprehensive emission control measures since 2013, which include (i) strengthening industrial and vehicle emissions standards; (ii) phasing out outdated industrial capacity, small high-emitting factories and small coal-fired industrial boilers; (iii) installing non-methane volatile organic compound emission control facilities; and (iv) replacing residential coal consumption with electricity and natural gas [3]. Most notably, the ‘ultra-low’ emission standard came into force in 2014 to control pollutions from coal-fired power plants. Consequently, the emissions of criteria air pollutants, such as fine particulate matter (PM_2.5_), SO_2_, NO*_x_* and CO, were successfully reduced by 21%–59% during 2013–2017 [3].

Hg can be toxic to human and ecosystem health. Hg in air is transported globally due to the long atmospheric lifetime (3 months to 2 years) of gaseous elemental mercury (GEM), the dominant form of atmospheric Hg [4]. GEM in the atmosphere can be deposited onto Earth's surfaces directly through dry deposition and indirectly through atmospheric oxidation followed by wet and dry deposition of Hg(II). On regional to global scales, direct GEM dry deposition contributes more than half of the total atmospheric Hg deposition [5]. The deposited Hg can be converted into methylmercury in aquatic ecosystems and subsequently bioaccumulated in food chains, posing severe health risks to humans and wildlife [6]. The Minamata Convention on Hg went into force in 2017, aiming to protect human health and the environment from anthropogenic releases of Hg. Considering that China has been the world's leading emitter of atmospheric Hg (∼30% of the world total) since the 1990s [1,7], the changes in anthropogenic Hg emissions and ambient GEM concentrations in China bear important implications for Hg budget and cycling in the global environment.

Due to the co-benefits of air pollution emission controls since the early 2010s [3], anthropogenic Hg emissions in China were expected to decrease after decades of continuous increase according to bottom-up inventory studies [8,9]. However, large uncertainties (equivalent to ±1 SD: −20% to 23%) exist in these estimates in terms of total emission amounts and associated temporal trends. Several studies reported appreciable declines in GEM concentrations (8%–22% yr^−1^) during 2014–2019 [10–12] in urban areas of eastern and northern China. These observations typically lasted for <5 years and are inadequate for exploring the long-term trends in ambient GEM concentrations and anthropogenic Hg emissions in China.

**Figure 1.**
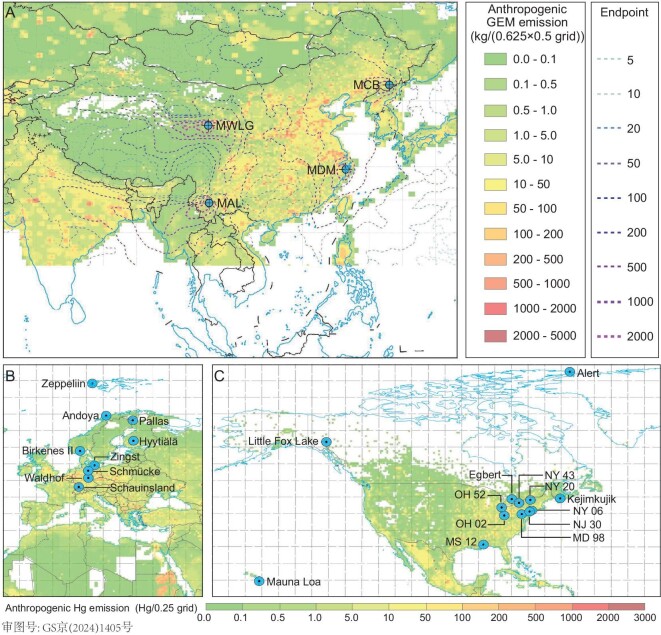
Locations of the long-term atmospheric GEM sampling sites. (A) Locations of rural sampling sites in China and anthropogenic GEM emissions in Asia in 2013 [1,8]. The dashed-line contours represent an example of the number of 5-day backward trajectory endpoints at the sampling sites during 2013. (B) Locations of the EMEP sites in Europe and the Polar regions. (C) Locations of the AMNet and CAPMoN sites in North America, the Polar regions and the Pacific Ocean. Color map in (B) and (C) shows the global gridded anthropogenic Hg emissions in 2015 [50].

In this study, we report results of the first-ever long-term observations (2008–2022) in GEM concentrations at four rural sites of different regions in mainland China (Fig. 1). The concentration trends are compared with those observed in other regions of the northern hemisphere. Analyses of criteria air pollutant data in China were performed to understand the drivers of the long-term GEM concentration trend. Machine-learning and empirical relationship models were applied to the observed GEM data to estimate the rate of anthropogenic GEM emission changes in China over the past decade.

## RESULTS AND DISCUSSION

### GEM concentration declines and source regions

**Figure 2.**
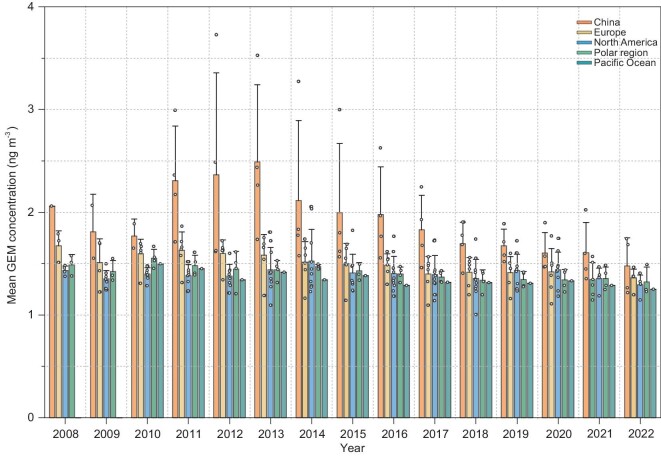
Comparison of annual mean GEM concentrations between China and other regions in the northern hemisphere. The white dots are the site-specific yearly mean GEM concentrations. Error bars represent the 1 SD of the mean GEM concentrations in the regions. GEM concentrations in Europe, North America, Pacific Ocean and Polar regions were obtained from EMEP [47], AMNet [48] and CAPMoN networks.

Multi-year mean ± 1 SD (based on annual mean) GEM concentrations at Mt. Waliguan (MWLG), Mt. Changbai (MCB), Mt. Ailao (MAL) and Mt. Damei (MDM) during the entire study periods were 1.85 ± 0.23 (annual mean in the range of 1.57–2.26, *n* = 15), 1.53 ± 0.14 (1.21–1.74, *n* = 14), 1.81 ± 0.40 (1.26–2.49, *n* = 12) and 2.57 ± 0.71 (1.74–3.72, *n* = 12) ng m^−3^, respectively (Fig. 2 and Table S1). The highest mean GEM concentration was observed at MDM in eastern China under the influence of strong regional anthropogenic sources and the lowest value occurred at MCB, possibly due to its being remote from source regions and the assimilation of GEM by vegetation (Fig. 1) [13,14].

In 2013, the annual mean GEM concentration ranged from 1.74 to 3.52 ng m^−3^ at the four Chinese sites with an overall mean of 2.49 ng m^−3^, which was ∼70% higher than the overall mean (1.47 ng m^−3^, range: 1.09–1.81) observed at other global network sites. Since 2013, the mean GEM concentration at the four Chinese sites decreased continuously to 1.48 ng m^−3^ in 2022, which was ∼12% higher than the mean value at other network sites (1.32 ng m^−3^, *n* = 15) in 2022 (Fig. 2 and Table S1). Noticeably, the annual mean GEM concentrations in 2022 at MCB (1.21 ng m^−3^) in northeastern China and MAL (1.26 ng m^−3^) in southwestern China had already fallen to within the range of 1.14–1.49 ng m^−3^ (*n* = 15) in Europe, North America, Polar regions and the Pacific Ocean, although those at MWLG (1.68 ng m^−3^) in northwestern China and MDM (1.74 ng m^−3^) in eastern China were still slightly higher than the upper-end values reported elsewhere.

### Trends in GEM concentrations

**Figure 3.**
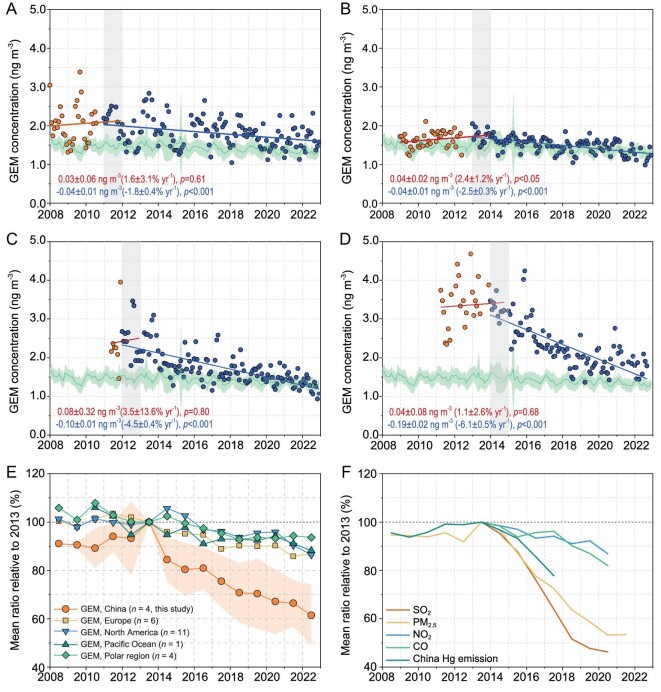
Trends of GEM and criteria pollutant concentrations. (A–D) Monthly mean GEM concentrations at MWLG, MCB, MAL and MAL, respectively. (E) Comparison of the trends in GEM concentrations in China, Europe, North America, Pacific Ocean and Polar regions during 2008–2022. (F) Trend in ground-level concentrations of air pollutants near the four sampling sites and anthropogenic Hg emissions in China during the study period. Light gray shaded areas in (A–D) indicate the trend reversal years. The green line and light-green shaded area are the monthly mean and 1 SD of the GEM concentrations observed elsewhere in the northern hemisphere, respectively. Data are normalized by dividing the means of each year by their corresponding means in 2013 in (E) and (F). The light-orange shaded area in (E) is the 1 SD of the GEM trend in China. GEM concentrations in Europe, North America, the Pacific Ocean and the Polar regions were obtained from EMEP [47], AMNet [48] and CAPMoN networks. Ground-level air pollutants data are from SDTF [29,49]. Chinese anthropogenic Hg emission data are from Liu *et al.* [8].

Applying the trend reversal method (see Text S1) to the long-term GEM concentration data identified a reversal year of 2011, 2013, 2012 and 2014 at MWLG, MCB, MAL and MDM, respectively (Fig. 3A–D). The increasing trends before the reversals were statistically insignificant at all sites except MCB, with the rates ranging from 1.1% to 3.5% yr^−1^ (*n* = 4). The upward trends in China contrast with the declines in GEM that were broadly observed in Europe, North America and the North Atlantic Ocean [15–18]. After the reversal year, GEM concentrations exhibited significant declines at a rate of 1.8%, 2.5%, 4.5% and 6.1% yr^−1^ (mean: 3.7% yr^−1^) at MWLG, MCB, MAL and MDM, respectively (*P*-values < 0.001 for all, Fig. 3A–D). The higher decreasing rates at MDM and MAL could have been a result of the more elevated annual mean concentrations (e.g. 3.27 ng m^−3^ at MDM in 2014 and 2.49 ng m^−3^ at MAL in 2012, Fig. 2) above the background level in the northern hemisphere in the reversal year, which provided a substantial potential for GEM concentrations to decrease in the process of the abatement of regional anthropogenic emissions. The decreasing rates of GEM in China were lower than those in Europe in the 1990s (e.g. Wank Mountain, Germany and Rörvik station, Sweden, from 7.0% to 9.0% yr^−1^) [19,20] when European anthropogenic Hg emissions were estimated to have decreased by 10% yr^−1^ [21], but were higher than the GEM decreasing rates observed worldwide since the 2000s (from 0.5% to 2.6% yr^−1^ in Europe, North America, East Asia and the North Atlantic Ocean) [15–17,22–25]. In the southern hemisphere, GEM concentrations showed a clear decrease (2.7% yr^−1^) from 1996 to 2009 [22], but remained relatively constant since 2009 [26].

A comparison of the long-term trends in GEM concentrations during 2008–2022 between the observations in China and elsewhere in the northern hemisphere is shown in Fig. 3E. From 2008 to 2013, GEM concentrations at the rural sites in China increased on average by 8.9%, whereas those in Europe and North America changed little (<4%) during the same period (Fig. 3E). From 2013 to 2022, GEM concentrations in China decreased by 38.6 ± 12.7%, which was much higher than the mean decreases in Europe (13.1%), North America (13.5%), Polar regions (6.4%) and the Pacific Ocean (11.8%) during the same period (Fig. 3E). The observations in China, together with those made elsewhere in the northern hemisphere, indicate a widespread decline in GEM concentrations in the northern hemisphere in the past decade. The declining GEM trends observed in China are consistent with the trends in anthropogenic Hg emissions during 2008–2017 (Fig. 3F), which also increased by 4.4% from 2008 to 2013, peaked in 2013 and then decreased by 22% from 2013 to 2017 [8].

### Anthropogenic emission reduction as the driver for GEM decline

A multiple-site concentration-weighted trajectories (CWTs) receptor model (see Text S2) reveals that the potential source regions of GEM at the four sites in China were consistent during 2013–2022, with relatively higher CWT values associated with air masses that originated from eastern (115–122°E, 25–35°N) and southwestern China (101–110°E, 25–35°N) (see Fig. S1 and Table S2) where major Hg anthropogenic sources are located [8]. A comparison of CWT values between 2013 and 2022 highlights the largest declines in eastern and southwestern China, with regional mean decline of 0.18 and 0.13 ng m^−3^ yr^−1^ in the GEM CWT values, respectively. This spatial feature mirrors the effectiveness of emission control measures for anthropogenic Hg emissions [8,9]. Relatively lower CWT values were mainly detected in air masses from Northeast Asia, north of Southeast Asia, Central Asia, the Tibetan plateau and Xinjiang Uyghur Autonomous Region in western China, where large anthropogenic Hg emission sources are absent.

**Figure 4.**
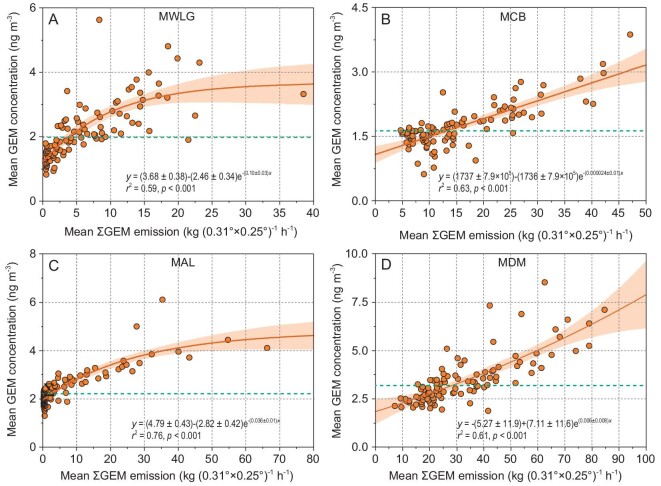
Relationship between the grouped mean GEM concentrations and corresponding mean cumulative exposure of air mass to Chinese anthropogenic GEM emissions at (A) MWLG, (B) MCB, (C) MAL and (D) MDM in 2013. GEM data are evenly divided into 100 groups with concentrations increasing from the lowest to the highest values at an interval of 1% percentile. The orange solid line and shaded area represent the exponential regression line and the 95% confidence area of the regression, respectively. The green dashed lines are the mean GEM concentrations at the sampling sites in 2013.

The effects of anthropogenic emissions in China on the variations in GEM concentrations were evaluated using the cumulative anthropogenic GEM emissions (ƩGEM emissions) encountered by the 120-h backward air mass trajectories in 2013 (see Text S3). ƩGEM emissions in China accounted for 96.9%, 88.6%, 54.0% and 97.3% of the total ƩGEM emissions in Asia at MWLG, MCB, MAL and MDM, respectively (Fig. S2). GEM concentrations at MWLG, MCB, MAL and MDM in 2013 positively correlated with ƩGEM emissions in China (*r*^2^ = 0.59 to 0.76, *P* < 0.001 for all, Fig. 4), but insignificantly correlated with ƩGEM emissions in the other Asian regions (Fig. S3), indicating that the variations in GEM concentrations at the four Chinese sites were predominantly controlled by domestic anthropogenic GEM emissions.

Long-term anthropogenic Hg emission inventories suggested that Hg emissions in China started to decrease during 2010–2013 after decades of increases and showed minimal temporal changes in the Hg emission speciation profiles over the past decade [8,9,27], mainly due to the co-benefits that resulted from the implementation of air pollution control devices (APCDs) for reducing PM_2.5_, SO_2_ and NO*_x_* emissions. As shown in Fig. 3E and F, the GEM trends in China during 2008–2022 were closely correlated with those of criteria air pollutants (PM_2.5_, SO_2_, NO_2_ and CO). Site-specific GEM trends significantly correlated with the trends of PM_2.5_ and SO_2_ emissions (except for one case between GEM and SO_2_ at MWLG). The GEM trends also correlated with trends of NO_2_ and CO at MDM and with trend of CO at MDM (Figs S4 and S5). We concluded that the recent declines in GEM concentrations in China were mainly caused by the control measures that were originally aimed at reducing anthropogenic PM_2.5_ and SO_2_ emissions, consistently with the results from earlier modeling work [8]. The declines in criteria air pollutants in recent years in China were primarily driven by the comprehensive implementation of APCDs [28,29], which synchronously reduced anthropogenic Hg emissions and led to lower GEM concentrations.

There were other factors that could potentially have contributed to the declines in GEM concentrations in China, although these factors were minor contributors, as discussed below (Table S3). (i) Changes in the large-scale and mesoscale atmospheric flow, which modulate the interannual variations in mean ƩGEM emissions, can influence the source–receptor relationship of atmospheric GEM. Yearly mean ƩGEM emissions at the four Chinese sites varied slightly from 2013 to 2022 (mean ratio relative 2013: 93.1%–113.9%) but did not show a similar consistent decline to that of GEM concentrations (Fig. S6), suggesting that the interannual variations in meteorology played a minor role in the long-term trends of GEM concentrations in China. (ii) A modeling study on atmospheric Hg mass balance suggested that Hg transport from other regions in the northern hemisphere contributed ∼51% of the GEM budget in East Asia [30]. Given the mean GEM decline of 0.16 ± 0.04 ng m^−3^ in Europe, North America, the Pacific Ocean and Polar regions from 2013 to 2022 (Fig. 2), the GEM declines in these regions would have contributed to a decline of only 0.08 ng m^−3^ in GEM concentrations in East Asia, which is <10% of the observed total declines in China from 2013 to 2022 (four-site mean of 1.01 ng m^−3^). (iii) Vegetation uptake of GEM plays a role in the long-term changes in GEM on a global scale [31]. The mean normalized difference vegetation index (NDVI) in the study areas continuously increased by 0.7% yr^−1^ from 2008 to 2022 (Fig. S4). Given the vegetation uptake of GEM of 100.4 tons in 2013 and the 5.7% increase in NDVI from 2013 to 2022 in China [32], the vegetation uptake of GEM in China would have increased by 5.7 tons during this period based on the assumption of the linear relationship between GEM uptake and NDVI. The increased vegetation uptake represents ∼2% of the total GEM deposition in China (250–280 tons yr^−1^, including direct uptake and atmospheric oxidation followed by wet and dry deposition of Hg(II)) [8,33], which is not likely to have caused a significant GEM concentration decline. (iv) Reemissions of previously deposited Hg are expected to decrease immediately and proportionally to anthropogenic emissions reduction [34,35] and could contribute to the long-term GEM trend [22,24]. Stable Hg isotope studies revealed that the reemissions of deposited Hg occurred immediately after deposition and decreased to nearly zero within 1–2 months, with an annual mean reemission quantity at 6%–14% of the deposited Hg (mean: 10% ± 2%, *n* = 8) [36–38]. From 2013 to 2017, the reduction in anthropogenic Hg emissions decreased atmospheric Hg deposition in China by ∼53 tons [8]. Using the reported reemission/deposition ratios, GEM reemissions from land surfaces in China were estimated to decrease by 5.3 ± 1.0 tons during 2013–2017, which is equivalent to 8% of the anthropogenic GEM emission reduction [8]. (v) Biomass burning in East Asia and Southeast Asia were of minor importance and did not show clear declines since 2013 [39]. (vi) The concentrations of OH and Br, which are the primary atmospheric GEM oxidants, also remained relatively constant in the global atmosphere during 2013–2022 [40,41]. These last two factors are therefore unlikely to have been responsible for the observed GEM declines in China.

### Estimates of the trends in China's anthropogenic GEM emissions

Previous estimates of the trends in China's anthropogenic Hg emissions based on bottom-up inventories varied significantly (Fig. S7) [8,9], largely because of the different methodology and varying removal efficiency application rates of the APCDs applied in these studies. Here we combined the GEM observations, air mass backward trajectories and gridded anthropogenic GEM emissions in 2013 from Liu *et al.* [8] to estimate the trends in China's anthropogenic GEM emissions during 2013–2022 using a machine-learning model (Convolutional Neural Network model, CNN) and an empirical relationship model (see Text S4 and S5).

**Figure 5.**
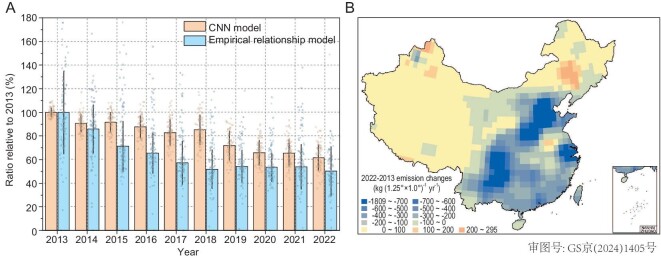
Temporal and spatial changes in China's anthropogenic GEM emissions during the past decade. (A) Temporal trends in China's anthropogenic GEM emissions during 2013–2022 derived from the CNN and empirical relationship model. Data are normalized by dividing the emissions in each year by the emissions in 2013. Light-orange dots are the optimal simulations selected by the two criteria in the CNN model (*n* = 90). Light-blue dots are the ratios estimated for the 100 groups in the empirical relationship model. (B) Spatial distribution of the 2022–2013 changes in China's anthropogenic GEM emissions simulated by using the CNN model.

Results from the CNN and the empirical model show 38% and 50% declines, respectively, in anthropogenic GEM emissions in China from 2013 (303 tons) to 2022 (187 and 152 tons) (Fig. 5A and Tables S4 and S5). Note that this analysis did not separate the contributions of the other factors to the observed GEM declines (i.e. reemissions, regional transport of background air mass and vegetation uptake in the empirical relationship model; and reemissions and regional transport of background air mass in the CNN model). However, these factors should have only played a minor role, as discussed above. Our estimates of the declines in China's anthropogenic GEM emissions during 2013–2022 (4.2%–5.5% yr^−1^) are overall consistent with those predicted by bottom-up inventories (3.4%–5.6% yr^−1^) [8,9].

The CNN model-inferred spatial distribution patterns of China's anthropogenic GEM emission sources during 2014–2022 are similar to those in 2013, with the dominant source regions located in northern and eastern China (Fig. S8). Large reductions in China's anthropogenic GEM emissions from 2013 to 2022 were mainly identified in northern (110–118°E, 32–41°N, 39 tons), eastern (118–122°E, 28–34°N, 13 tons) and southwestern China (100–110°E, 23–33°N, 36 tons) (Fig. 5B)—a finding that is closely aligned with previous inventory estimates [8,9]. Results from the CNN and empirical relationship model showed that the decreasing rates of anthropogenic GEM emissions were higher from 2013 to 2019 (by 4.7% and 7.6% yr^−1^, respectively) and then became lower from 2019 to 2022 (by 1.3% and 3.4% yr^−1^, respectively) (Fig. 5A). Such a trend is consistent with those of surface-level PM_2.5_ and SO_2_ concentrations in China, with a decline of 6.9% yr^−1^ and 8.7% yr^−1^, respectively, during 2013–2019 and by 2.6% yr^−1^ and 1.5% yr^−1^, respectively, during 2019–2022 (Fig. 3F). The lower declines after 2019 could have been a result of the limited potential for further implementation of emission control measures [8] and the increased industrial activities (Fig. S9).

## CONCLUSIONS AND ENVIRONMENTAL IMPLICATIONS

This study reports significant declines in GEM concentrations in Chinese rural areas since the early 2010s, reflecting the effectiveness of China's Clean Air Action on reducing domestic anthropogenic Hg emissions. We estimate that anthropogenic GEM emissions were reduced by 116–151 tons from 2013 to 2022. Given the global anthropogenic GEM emissions of 1527–1725 tons during 2010–2015 [1], the reductions in China represent a 7%–10% drop in the global GEM emissions. Because of the highly elevated background GEM concentrations in East Asia, long-range transport of GEM from this region (mainly China) has been regarded as an important source of atmospheric GEM and Hg deposition in the other regions of the northern hemisphere, e.g. contributing 10%–37% (0.16–0.80 ng m^−3^) of atmospheric GEM concentrations and 15%–33% (3.0–4.4 μg m^−2^ yr^−1^) of the Hg depositions in Europe, North America and Polar regions [30,42–44]. We estimate that the 38.6% decline in ground-level GEM concentrations in China contributes to a 4%–14% decline in GEM concentrations and 6%–13% decline in Hg deposition in Europe, North America and Polar regions, which could partially explain the observed declines in GEM concentrations in these regions during 2013–2022.

Our results also indicate that abatement of anthropogenic emissions could reduce ground-level GEM concentrations more effectively than previous model-predicted results showed. For example, Liu *et al.* predicted that a 22% reduction in national anthropogenic emissions resulted in a 11% decline in GEM concentrations in China from 2013 to 2017 [8], which was lower than the result (∼19%) obtained in the present study (Fig. S10). In addition, a previous global modeling study showed that a 30%–35% decline in GEM concentrations in Europe and North America from 1990 to 2010 required a 73%–85% reduction in anthropogenic GEM emissions in these regions (reduced from 399–410 to 61–109 tons) [16], whereas a similar magnitude of GEM decline in China required a 35%–41% reduction in anthropogenic GEM emissions (Fig. S10). We therefore suggest that anthropogenic emissions play a more important role in the regional atmospheric GEM budget than traditionally thought, likely because the natural emissions and inflows of GEM from other regions in the northern hemisphere are overestimated. Alternatively, the decreasing trends in Chinese anthropogenic GEM emissions estimated in this and the previous studies are underestimated. Future modeling studies should incorporate long-term continuous observations worldwide to better evaluate the effectiveness of the Minamata Convention and understand the cycling of atmospheric Hg at the global scale.

## MATERIALS AND METHODS

### Sites description

Four sites representing different geographical regions across China were selected for investigating the temporal trends in GEM concentrations in China (Fig. 1). Mt. Waliguan (MWLG, 100.898°E, 36.287°N, 3816 m a.s.l.) is located in the northeast of the Tibetan Plateau in northwestern China. Mt. Changbai (MCB, 128.112°E, 42.402°N, 741 m a.s.l.) is in a temperate forest in northeastern China. Mt. Ailao (MAL, 101.020°E, 24.533°N, 2450 m a.s.l.) is located in a subtropical forest in southwestern China. Mt. Damei (MDM, 121.565°E, 29.632°N, 550 m a.s.l.) is in eastern China, close to the East China Sea. These sites are isolated from local anthropogenic emissions and receive air masses that pass over nearly all the geographical regions of China (also including the major anthropogenic Hg source regions in China, Fig. 1). Therefore, monitoring data at these sites were analysed to study the trends in atmospheric GEM concentrations and estimate the changes in anthropogenic Hg emissions in China.

### Measurements of GEM concentrations

GEM concentrations at MWLG, MCB, MAL and MDM were continuously measured using Tekran 2537 A/B/X automated Hg analysers since September 2007, October 2008, June 2011 and April 2011, respectively. MWLG, MCB and MAL were part of the monitoring network of the Global Mercury Observation System (GMOS). The GMOS Standard Operating procedures were followed for the GEM measurements at all four sites [45]. Tekran analysers were operated at a mass flow rate (referenced to 0°C and 760 mmHg of pressure) of 0.5–0.75 L min^−1^ and a sampling interval of 10 min at MWLG, 0.9–1.0 L min^−1^ and 5 min at MCB, 0.75 L min^−1^ and 5 min at MAL and 1.0 L min^−1^ and 5 min at MDM. Tekran analysers were routinely and automatically calibrated using the internal permeation sources at intervals of 47–71 h and the permeation rates of internal sources were periodically validated by injections of known amounts of Hg^0^ vapor every several months. Co-located measurements of GEM concentrations using the Tekran analysers at the four Chinese sites showed mean deviations in the range of 1%–4% (*n* = 3). Concentrations of Hg measured by using the Tekran analysers only contained small fractions (0.1%–0.4%) of gaseous oxidized mercury at our sampling sites [46] and therefore the measured Hg was designated as GEM.

### Long-term GEM data outside China and criteria air pollutants and NDVI data in China

GEM concentrations at 6 sites in Europe, 11 sites in North America, 4 sites in the Polar regions and 1 site in the free troposphere of the Pacific Ocean during 2008–2022 (Fig. 1 and Table S6) were obtained from the European Monitoring and Evaluation Programme (EMEP) database (https://ebas.nilu.no/) [47], Atmospheric Mercury Network (AMNet, https://nadp.slh.wisc.edu/networks/atmospheric-mercury-network/) [48] and Canadian Air and Precipitation Monitoring Network (CAPMoN, https://open.canada.ca/en). Ground-level concentrations of criteria air pollutants at 3° × 3° resolution at the four sampling sites in China were obtained from the Satellite Data to Freshman (SDTF, https://weijing-rs.github.io/product.html) [29,49]. NDVI at 3° × 3° resolution at the four sampling sites in China during 2008–2022 are from the NASA Earth Observations platform (https://neo.gsfc.nasa.gov/).

### Additional methods

Additional methods for backward air mass trajectory, CWTs receptor model, trend reversals detection and exposure of air masses to anthropogenic Hg emissions (ƩGEM emissions) are presented in the Supplementary Data. A CNN machine-learning model and an empirical relationship model were applied to estimate the changes in anthropogenic GEM emissions in China over the past decade. The CNN machine-learning model established a source–receptor relationship matrix between gridded GEM CWTs values and anthropogenic emissions, natural surface emissions (or sinks) and the transport of background GEM in 2013, which allowed prediction of the spatial distributions of anthropogenic GEM emissions in China during 2014–2022. The empirical relationship model used the exponential regression between ƩGEM emissions in China and measured GEM concentrations established in 2013 to estimate the reduction rates of Chinese anthropogenic GEM emissions during 2014–2022 relative to 2013. Results from the CNN machine-learning model were corroborated by the empirical relationship model to constrain the trends in Chinese anthropogenic GEM emissions over the past decade. More information on the CNN machine-learning and empirical relationship model is presented in the Supplementary Data.

## Supplementary Material

nwae264_Supplemental_Files

## Data Availability

All data are available in the main text, Supplementary Data and Supplementary Data Tables. GEM concentrations data at the four investigated sites in China during the whole study periods are available from the corresponding author upon request.
